# Greening of human-dominated ecosystems in India

**DOI:** 10.1038/s43247-023-01078-9

**Published:** 2023-11-27

**Authors:** Taejin Park, Murali K. Gumma, Weile Wang, Pranay Panjala, Sunil K. Dubey, Ramakrishna R. Nemani

**Affiliations:** 1 NASA Ames Research Center, Moffett Field, California USA; 2 Bay Area Environmental Research Institute, Moffett Field, California USA; 3 International Crop Research Institute for Semi-Arid Tropics, Patancheru, Telangana India; 4 Mahabalonis Crop Forecasting Center, Pusa, Delhi, India

**Keywords:** Environmental impact, Agroecology

## Abstract

Satellite data show the Earth has been greening and identify croplands in India as one of the most prominent greening hotspots. Though India’s agriculture has been dependent on irrigation enhancement to reduce crop water stress and increase production, the spatiotemporal dynamics of how irrigation influenced the satellite observed greenness remains unclear. Here, we use satellite-derived leaf area data and survey-based agricultural statistics together with results from state-of-the-art Land Surface Models (LSM) to investigate the role of irrigation in the greening of India’s croplands. We find that satellite observations provide multiple lines of evidence showing strong contributions of irrigation to significant greening during dry season and in drier environments. The national statistics support irrigation-driven yield enhancement and increased dry season cropping intensity. These suggest a continuous shift in India’s agriculture toward an irrigation-driven dry season cropping system and confirm the importance of land management in the greening phenomenon. However, the LSMs identify CO_2_ fertilization as a primary driver of greening whereas land use and management have marginal impacts on the simulated leaf area changes. This finding urges a closer collaboration of the modeling, Earth observation, and land system science communities to improve representation of land management in the Earth system modeling.

## Abstract (web summary)

Satellite-derived leaf area and survey-based agricultural data indicate that dry season irrigation contributes to India’s greening, however land surface models struggle to accurately reproduce the greening trend and its drivers.

## Introduction

Long-term satellite observations show the Earth has been greening (increasing greenness or green leaf area)^1–6^. This global scale land surface change can lead to significant consequences in the Earth’s energy, water, and carbon cycles^5^, and has been recognized as highly credible evidence of intensified terrestrial biosphere activity in response to anthropogenic climate change^7^. Recent studies attributed the satellite-observed vegetation changes to environmental drivers and identified CO_2_ fertilization, climate change, nitrogen deposition, and land cover/use changes (LCLUC) as underlying drivers of global greening in the order of contribution^5,8–10^. Despite the consensus on the potential role of CO_2_ fertilization on the observed greening, a comprehensive understanding of the satellite-observed vegetation changes and their associated mechanisms is still lacking and debated^6,11,12^.

One of the most important but understudied drivers of global greening is human land use and management, especially over global croplands^6^. Cropland is the most pervasive anthropogenic biome occupying approximately 1244 Mha (about 9.5% of ice-free land mass) of the Earth’s land surface^11^. Studies based on satellite remote sensing have identified six global breadbaskets as greening hotspots: India, USA, Canada, Europe, Brazil, and sub-Saharan regions^6,13,14^. This prominent greening of croplands underscores the overlooked role of human land use and management in global greening research, thereby necessitating a comprehensive investigation into how land management practices have triggered a significant increase in leaf area on a large scale. Notably, special attention should be directed towards investigating Indian croplands, as they alone contribute to over 11% of the total net increase in global leaf area and these croplands exhibit the most extensive greening, encompassing 69% of the total vegetated regions in India^6^. Given the challenges faced by India’s agricultural system due to changing climate and water resources, it becomes crucial to understand the underlying drivers of this cropland greening. Such understanding will enable us to better project future changes in Indian agriculture systems and consequently prepare for ensuring food security.

Agriculture in India is the primary source of livelihood for more than 58% of India’s 1.4 billion population and it thus plays important roles in the social and political economy^15^. There are two dominant cropping seasons in India based on monsoon, i.e., Kharif (June–October, wet season) and Rabi (November–May, dry season). The Kharif season crops (e.g., rice, maize, etc.) are grown with the onset of monsoon and generally require more water. Crops in the Rabi season (e.g., wheat, barley, etc.) are generally sown in winter and harvested before monsoon season. Rabi crops need cold weather for growth and need less water than the Kharif crops. Despite the lower water demands of Rabi crops, drier Rabi season without monsoon necessitates substantial water inputs requiring an irrigation-assisted cropping system. Irrigation stands as India’s most crucial land management practice in agriculture since the Green Revolution, serving as a tool to alleviate drought stress and thus increase crop yield and production^16,17^. Notably, the country boasts the world’s largest irrigated area^18^. This irrigation-associated crop yield enhancement can be anticipated as a key driver in the observed Indian cropland greening^19^. Yet, the spatiotemporal dynamics of how irrigation has changed the satellite-observed greenness as well as the national crop yield remains unclear. Subsequently, the degree to which land surface models (LSMs), which have been used for the greening attribution studies, accurately represent the changes in leaf area associated with human land management is not fully understood.

In this study, we formulate and address the following research question: *What is the role of irrigation as a human land management practice in the observed greening patterns in Indian croplands?* We incorporate satellite remote sensing and survey-based Indian agriculture statistics to investigate the satellite-observed greening patterns and explore the underlying mechanisms. We further utilize the results from experiments using the state-of-the-art dynamic global vegetation models (DGVMs) factorial simulations to attribute the leaf area changes to underlying drivers and examine the identified roles through comparisons to the satellite measurements and national statistics.

## Results

### Rapid dry season greening in India

**Fig. 1:**
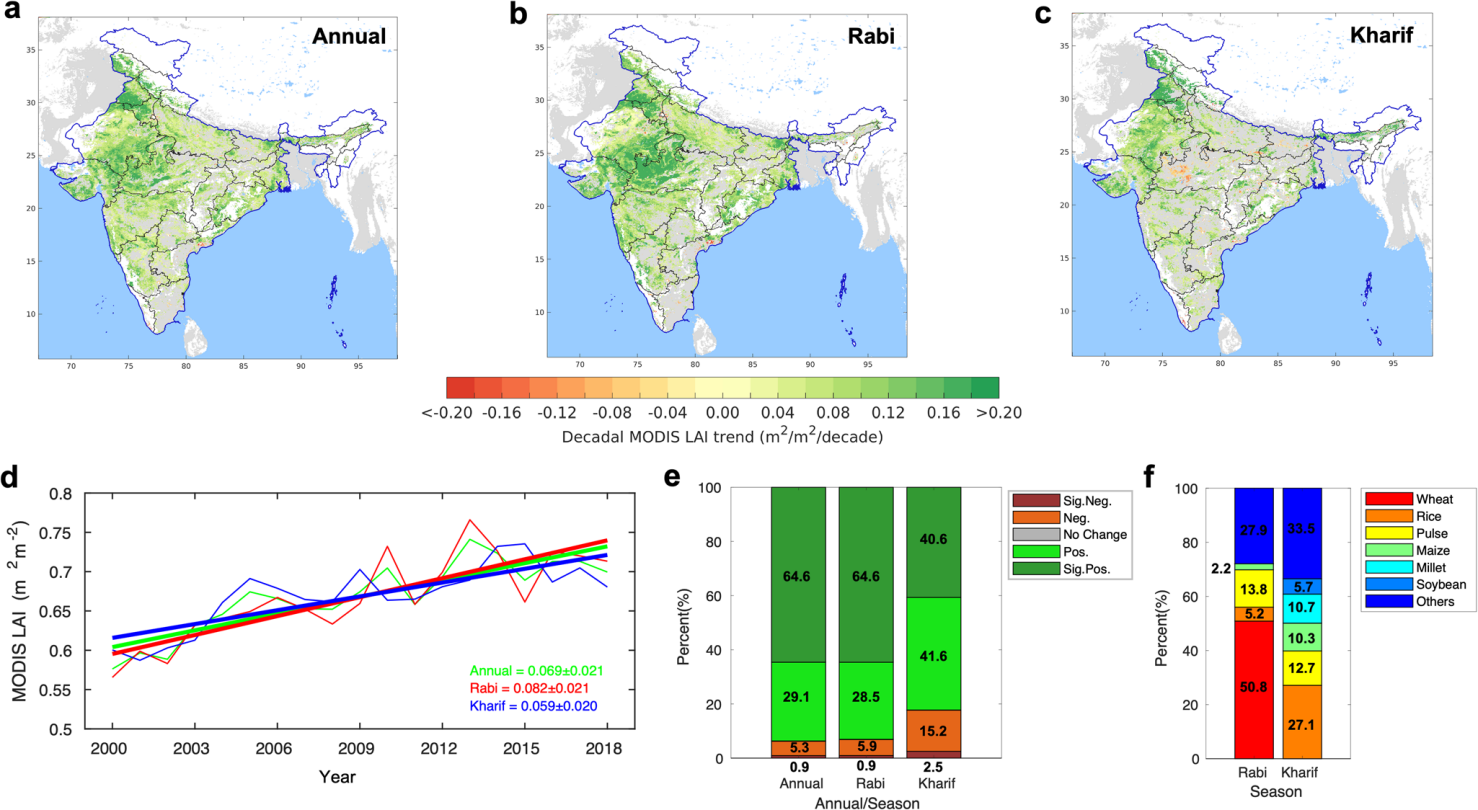
Changes in MODIS leaf area index (LAI) over 2000–2018. **a** Spatial pattern of decadal trends in annual average MODIS LAI over Indian croplands. Statistically significant trends (Mann–Kendall test, *p* < 0.1) are color-coded. Gray areas show croplands with statistically insignificant trends. White areas depict non-croplands including forests, barren lands, permanent ice-covered areas, permanent wetlands, and built-up areas. Black and blue lines are state and country boundaries. **b** Same as **a** but for Rabi season (November– May, dry season). **c** Same as **a** but for Kharif season (June–October, wet season). **d** Time series of annual (green), Rabi (red), and Kharif (blue) season LAI change. **e** Percent of greening and browning across Indian croplands in the annual, Rabi, and Kharif seasons during the last two decades (2000−2018). Red, orange, gray, light green, and dark green stand for significant negative, insignificant negative, no change, insignificant positive, and significant positive trends. Here *p*-value 0.1 is used for defining statistically significant trends. **f** Crop types over the areas showing significant positive LAI trends during Rabi (64.6% of total cropland areas) and Kharif (40.6% of total cropland areas) seasons.

We use 19 years of NASA Terra and Aqua Moderate Resolution Imaging Spectroradiometer (MODIS) leaf area index (LAI) data to detect changes in greenness over India’s croplands (Methods). During the last two decades, the annual MODIS LAI record shows a widespread greening (increasing) trend across Indian croplands (Fig. 1). The observed greening is prevalent over northwestern provinces, especially, Punjab, Haryana, Rajasthan, Gujarat, and Madhya Pradesh (Fig. 1a). About 64.6 % of the Indian croplands reveal a greening trend (*p* < 0.1) whereas 0.9 % of the croplands display a browning (decreasing, *p* < 0.1) trend. The rate of annual LAI change at the national scale is estimated by 0.069 ± 0.021 m^2^ ∙ m^-2^∙decade^-1^ (*p* < 0.001). The spatial patterns of the observed increasing green leaf area in the Rabi (dry) and Kharif (wet or monsoon) seasons are noticeably different (Fig. 1b, c). For instance, central India (Madhya Pradesh) displays a strong increase of LAI (0.131 ± 0.031 m^2^ ∙ m^-2^∙decade^-1^) during the Rabi season whereas a weak increase of LAI (0.022 ± 0.019 m^2^ ∙ m^-2^∙decade^-1^) during the Kharif season. Punjab, known as the most fertile region in India, shows a strong greening signal in both the Rabi and Kharif seasons. Overall, LAI change in the Indian croplands during the Rabi season is 0.082 ± 0.021 m^2^ ∙ m^-2^∙decade^-1^ (*p* < 0.001) and it is 38% greater magnitude of change compared to the Kharif season LAI change (0.059 ± 0.020 m^2^ ∙ m^-2^∙decade^-1^, *p* < 0.001) (Fig. 1d). The observed greening extent of the Rabi season LAI (64.6 % of the cropland) largely overlaps with the patterns of annual LAI change, and it is much larger than the Kharif season greening (40.6% of the cropland) (Fig. 1e). Note that about 2.5 % of the croplands display a decreasing LAI trend during the Kharif season while only 0.9 % of the regions exhibit declining LAI trends in the Rabi season. Our analysis further differentiates to what extent the seasonally varying greening in Indian cropland is associated with different crop types (Fig. 1f). We find that wheat is predominantly responsible for half of the cropland areas exhibiting the Rabi season greening (64.6% of total cropland area), while rice (27.1%), pulse (12.7%), millet (10.7%), and maize (10.3%) are associated with 61% of the Kharif season greening area (40.6% of total cropland area). The greening over wheat-cropped areas during the Rabi season consistently emerges as a strong greening region in terms of annual-scale LAI changes (Fig. 1a, b). These results from satellite observations indicate that the dry season greening is more prevalent and dominates overall (annual) Indian cropland greening during the last two decades.

### Role of irrigation in dry season greening

**Fig. 2:**
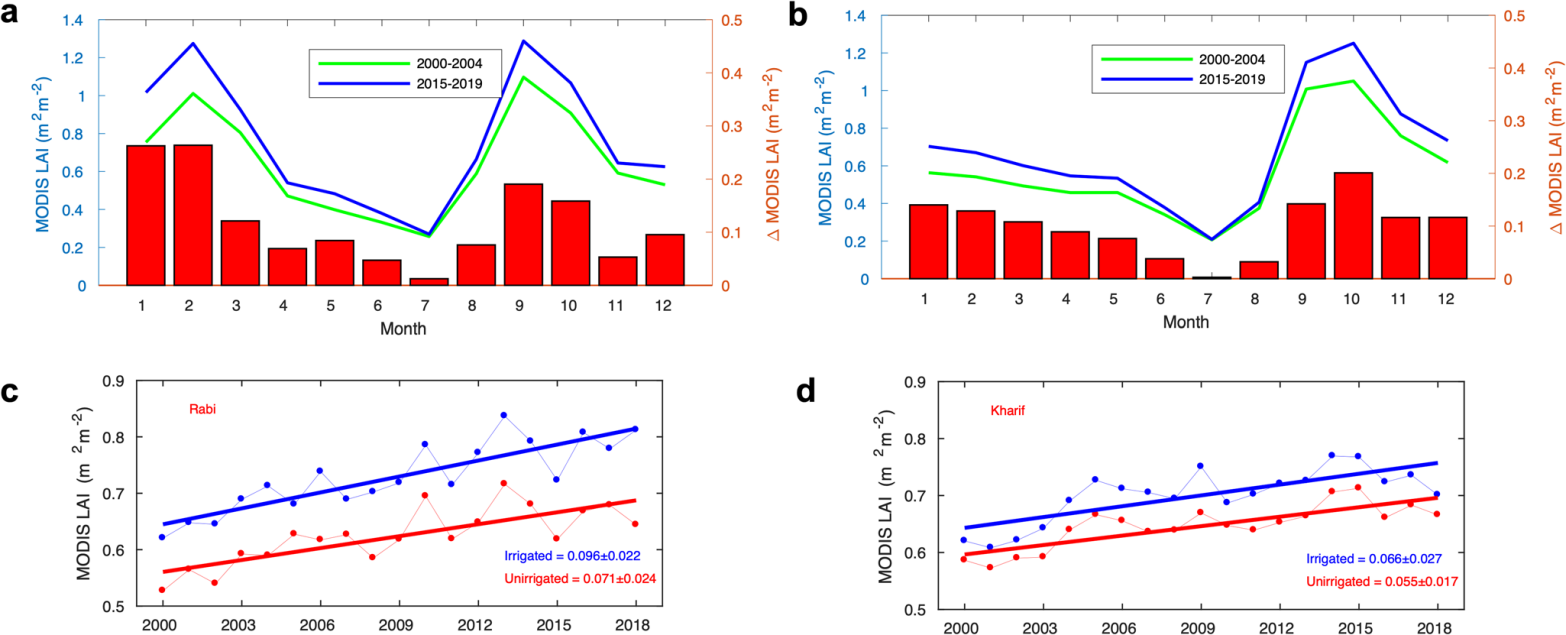
Changes in seasonal MODIS LAI in irrigated and unirrigated Indian croplands. **a** Seasonal variation of LAI in the irrigated Indian croplands. LAIs in two separate periods (green line: first 5 years, blue line: last 5 years) and their monthly differences (red bar) are plotted. **b** Same as **a** but for the unirrigated croplands. **c** Time series of Rabi-season averaged LAIs over the irrigated (blue) and unirrigated (red) croplands. **d** Same as **c** but for the Kharif season.

The role of irrigation in modulating leaf area changes over Indian croplands is evaluated using irrigated and unirrigated area maps^18^ (Supplementary Fig. 1). According to the irrigation maps, 87.8 Mha of the Indian croplands is irrigated. Indo-Gangetic Plain and foothills of Himalayas are the primary irrigated croplands. Over croplands across India, we observe distinct and different LAI seasonality over irrigated and unirrigated regions (Fig. 2a, b). Monthly composited MODIS LAI data reveals that irrigated croplands generally have double cropping systems and show higher LAIs in both dry (Rabi) and wet (Kharif) seasons, whereas the unirrigated (rainfed) regions display a single peak seasonality during the wet monsoon season indicating a primary cropping activity during a year. This difference in seasonality indicates an irrigation-driven increase in cropping intensity in Indian croplands^20,21^. There are other noticeable differences in seasonal changes of LAIs (Δ MODIS LAI in Fig. 2a, b) between areas irrigated and unirrigated. While we observe comparable increases of seasonal leaf area over both irrigated and unirrigated areas during the Kharif season, in the Rabi season, we find a greater degree of LAI increase over irrigated croplands compared to the unirrigated lands. Our time series analysis accords with these patterns (Fig. 2c, d). It indicates a comparable degree of LAI increase during the Kharif season (June–October) over both irrigated (0.066 ± 0.027 m^2^ ∙ m^-2^∙decade^-1^, *p* = *0.002*) and unirrigated (0.055 ± 0.017 m^2^ ∙ m^-2^∙decade^-1^, *p* < 0.001) croplands. However, the increase of LAI in the Rabi season (November–May) is 35 % greater than the LAI increase in the Kharif season: irrigated (0.096 ± 0.022 m^2^ ∙ m^-2^∙decade^-1^, *p* < 0.001) and unirrigated (0.071 ± 0.024 m^2^ ∙ m^-2^∙decade^-1^, *p* < 0.001). Wheat is the most extensively irrigated crop type in India’s agriculture. About 95% of the total wheat cropping area has been irrigated. Our results shown in Fig. 1f indicate that most of the greening during the Rabi season is linked to regions where wheat is cultivated. This independent evidence further bolsters the argument for irrigation-driven greening in India. Note that both irrigated and unirrigated croplands share similar interannual variation during dry and wet seasons suggesting a large-scale response of croplands to strong climate variability (e.g., 2015–16 drought)^22^.

**Fig. 3:**
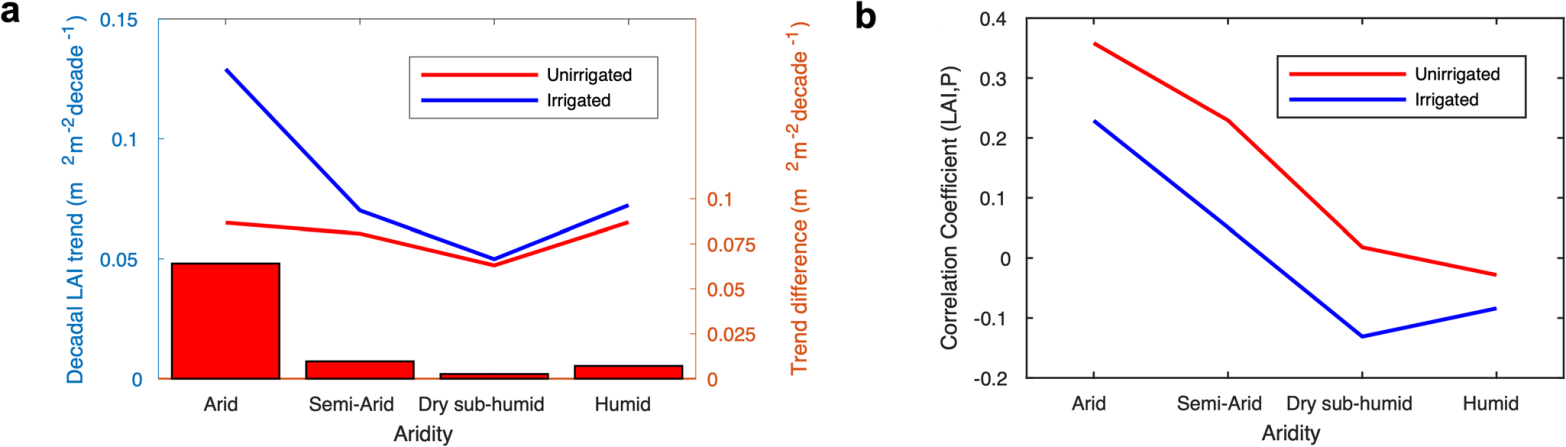
Effectiveness of irrigation on MODIS LAI changes over different aridity levels. **a** Decadal LAI trends over irrigated (blue line) and unirrigated (red line) croplands by different aridity levels, i.e., arid, semi-arid, dry sub-humid, and humid. Red bar represents the difference in LAI trends between irrigated and unirrigated croplands. **b** Correlation coefficient estimates between LAI and precipitation at different irrigation practices and aridity levels. The correlation coefficient is calculated by partial-correlation analysis with other climate variables (temperature and solar radiation).

We hypothesize that irrigated croplands are more resistant to water stress (or variability) so that the increases in LAI associated with irrigation are greater in drier regions^16,17,23^. We categorize croplands based on aridity levels (Supplementary Fig. 1a, arid, semi-arid, dry sub-humid, and humid; see Methods) to investigate the role of irrigation in the satellite-observed greening over Indian croplands (Fig. 3). We find that irrigation assisted croplands display the greatest increase of LAI over the arid environment where aridity value (i.e., ratio between precipitation and evapotranspiration) is lower than 0.2. and its degree of increasing trend is gradually declining in less-water-limited environments (Fig. 3a). In contrast, unirrigated croplands do not show statistically meaningful differences in the decadal LAI trends over varying aridity levels. We use the difference between LAI trends in irrigated and unirrigated croplands as an indicator of irrigation effectiveness. Our analysis confirms that irrigation is highly effective in arid environments. The trend estimates over the arid regions show a two times higher rate of LAI changes over irrigated croplands compared to unirrigated croplands. There are negligible trend differences observed in dry sub-humid and humid environments. Further analysis evaluating the dependence of LAI changes on precipitation variability confirms a less sensitive response of irrigated regions to rainfall variability compared to the unirrigated croplands. It implies a higher effectiveness of irrigation in promoting cropland greenness over water-limited environments.

### Shift in Indian cropping system

**Fig. 4:**
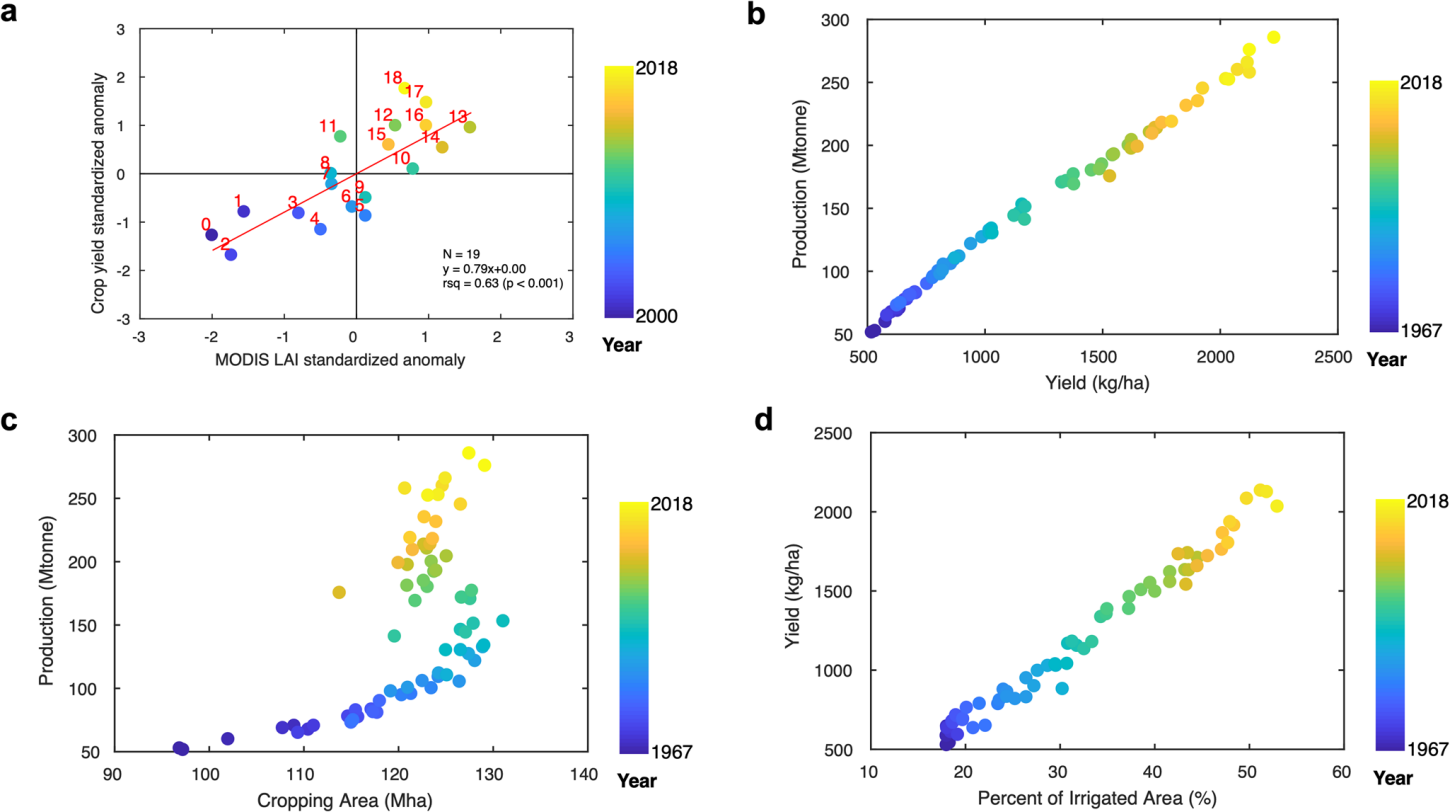
Historical changes in yield, production, cropping area, and percent of irrigated area in India. **a** Observed co-variations between standardized MODIS LAI and crop yield anomalies. Colored scatters stand for the year (2000–2018). **b** Changes and co-variation of crop production and yield. Colored scatters stand for the year (1967-2018). **c** Same as **b** but for cropping area and production. **d** Same as **b** but for yield and percent of irrigated area.

We further examine linkages between satellite remote sensing and independent survey-based Indian agriculture statistics to ensure the satellite-observed greening patterns and understand their underlying mechanisms. We first compare annual LAI to annual yield estimates (Fig. 4a). Our comparison indicates that historical MODIS LAI data can capture national statistics of annual crop yield (*R*^2^ = 0.63) conforming to the continuous increasing greenness and yield trends during the last two decades. This agreement supports that remote sensing-based national crop yield estimation is feasible and the observed spatial pattern of greening over the Indian croplands is valid. Indian agricultural statistics reveal a strong positive co-variation (*R*_corr_ = 1.00, *p* < 0.001) between total production (36.51 ± 1.50 MT∙decade^-1^, *p* < 0.001) and yield (293.46 ± 13.05 kg∙ha^-1^∙decade^-1^, *p* < 0.001), both exhibiting an upward trend from 1960s onwards (Fig. 4b). While the increases in cropping area played an important role in shaping the correlation (*R*_corr_ = 0.95, *p* < 0.001) between 1960s and 1980s, their contribution later became weaker (*R*_corr_ = –0.15, *p* = 0.339) between 1990s and 2010s (Fig. 4c). As total production is a function of yield and cropping area, strong coupling between irrigation and yield (*R*^2^ = 0.98) indicates a significant role of irrigation in increasing crop yield and total crop production in India (Fig. 4d). A one percent increase in irrigated area is estimated to increase crop yield by 43.17 ± 0.77 kg∙ha^-1^. This strong linkage between irrigation and crop yield supports a water-driven crop yield enhancement in India^16,24^.

**Fig. 5:**
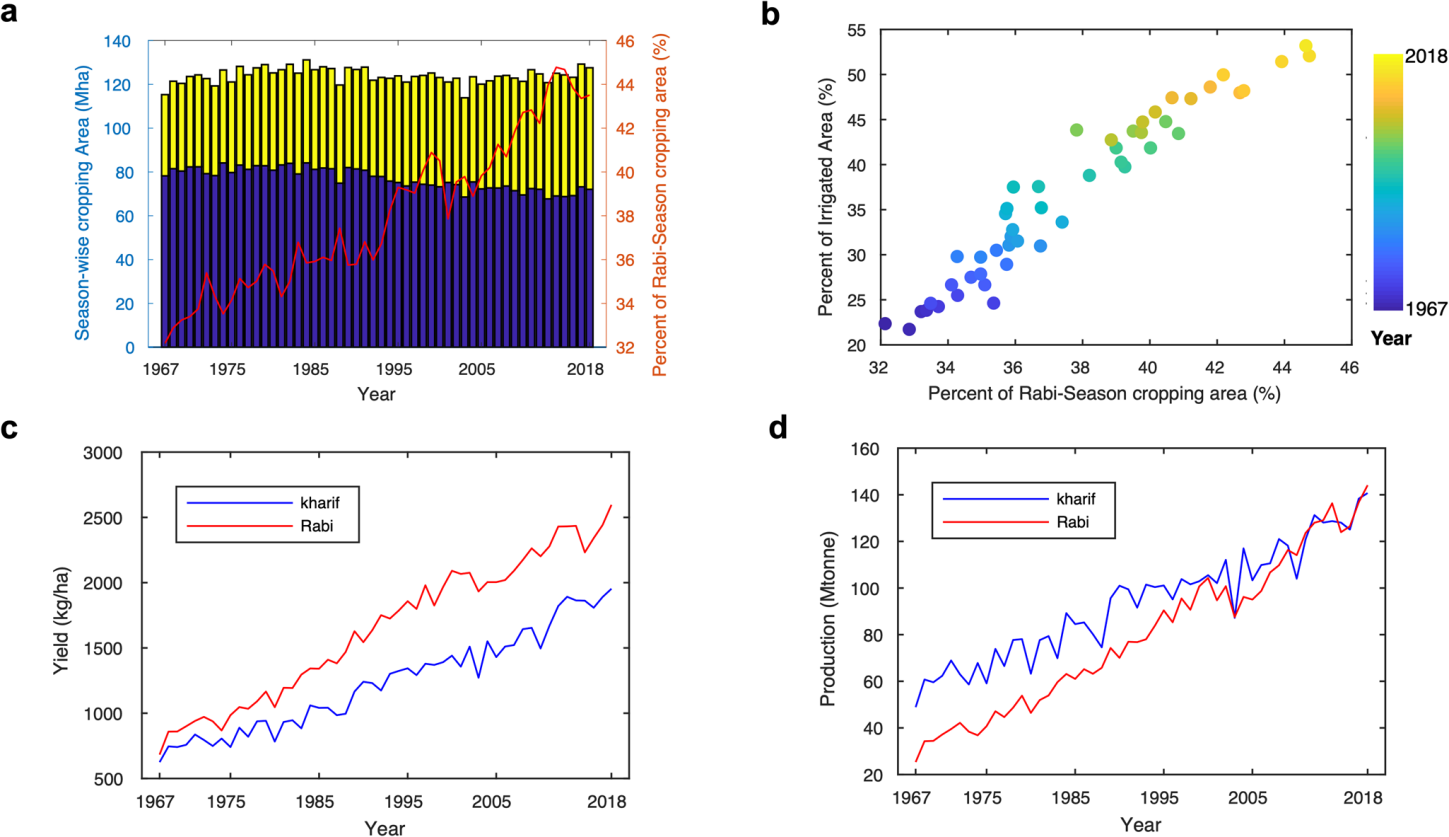
Enhanced Rabi season (dry season) crop yield and production through extensive irrigation practice. **a** Changes in total areas of Rabi (yellow bar) and Kharif (blue bar) season cropping, and percent of Rabi season cropping area (red line) during the last six decades (1967−2018). **b** Relation between percent of irrigated area and percent of Rabi season cropping area. Colored scatters stand for the year. **c** Changes in Rabi (red) and Kharif (blue) season crop yields. **d** Changes in Rabi (red) and Kharif (blue) season total production.

India has maintained stable total cropping areas (~120 Mha) during the last five decades but contrasting changes of the relative proportions of Rabi and Kharif season cropping areas have been observed (Fig. 5a). In the 1960s, the Rabi-season cropping area was responsible for ~32% of total cropping areas in India. Continuous increase of the Rabi-season portion (2.77 ± 0.24 Mha∙decade^-1^, *p* < 0.001) reached 44% of total cropping areas in the 2010s, whereas the Kharif season cropping area gradually declined over time (⎯2.84 ± 0.42 Mha∙decade^-1^, *p* < 0.001) indicating a significant expansion of Rabi-season cropping area (2.21 ± 0.13 %∙decade^-1^, *p* < 0.001) in India. The observed expansion of the Rabi-season cropping area is closely associated with the increasing irrigation practices (Fig. 5b). These changes lead to the significant enhancement of crop yield and total grain production during the Rabi-season (Fig. 5c). We find that the rate of increasing yield in the Rabi season (353.09 ± 13.16 kg∙ha^-1^∙decade^-1^, *p* < 0.001) is 45% greater than the rate in the Kharif season (242.09 ± 12.360 kg∙ha^-1^∙decade^-1^, *p* < 0.001). It is worth noting that recent total production in the Rabi season surpasses Kharif’s total production (Fig. 5d). This is a noticeable change because total grain production during the Rabi season was only half of the Kharif season production in the 1960s.

Contrasting crop-specific changes in cropping areas and irrigation practices are observed in India (Supplementary Table 3). For instance, the cropping area of wheat (Rabi season crop) has increased by 0.26 Mha∙decade^-1^, whereas the aerial extents of nutrient cereals and jowar (Kharif season crop) have decreased by –0.47 and –0.27 Mha∙decade^-1^, respectively. Though we see diminished cropping areas in some crop types, a significant increase in crop yield together with tight association with growing irrigation practices is prevalent across all major crop types in India (Supplementary Tables 3 and 4).

### Earth system models under-represent irrigation-driven greening in India

**Fig. 6:**
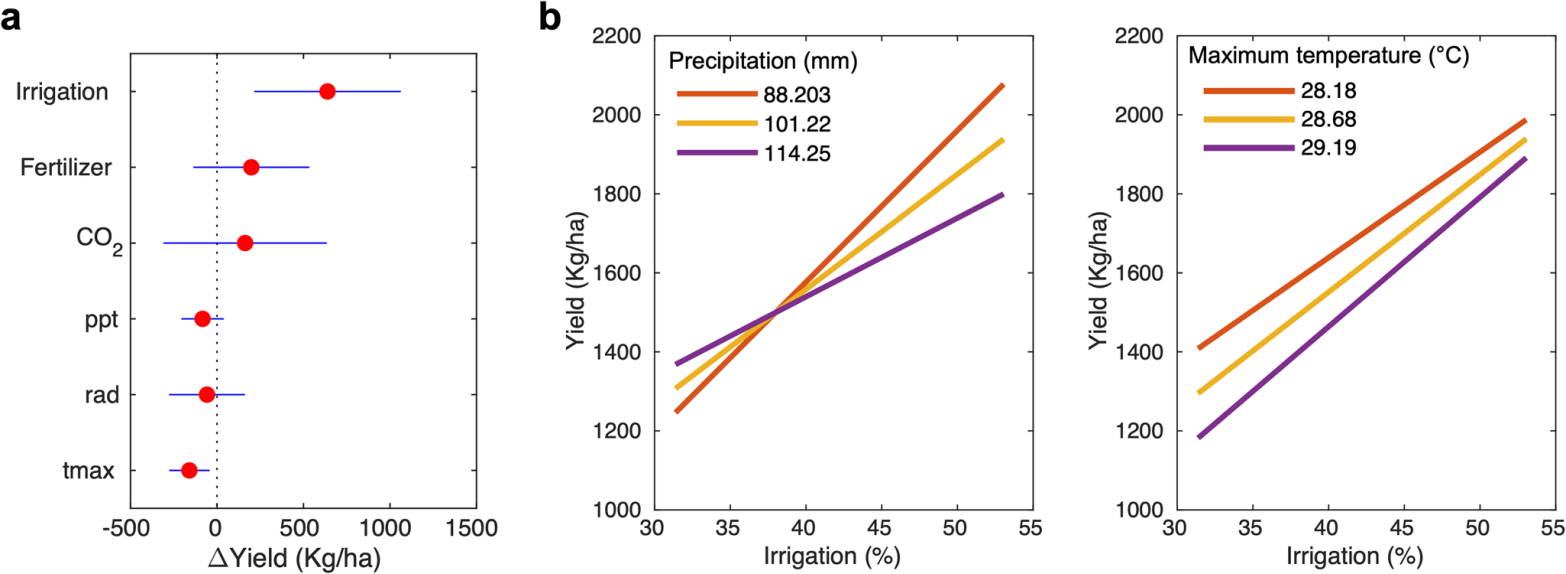
Drivers of historical crop yield changes in India. **a** Total effect size of irrigation, fertilizer use, CO_2_, precipitation, radiation, and maximum temperature on the observed changes in historical crop yield. **b** Interaction of irrigation and climate conditions on crop yield (left: precipitation, right: maximum temperature). Note that we use the 1985−2017 period of the data for this analysis due to the shorter period of fertilizer use record.

In addition to the first-order investigation, our multivariate statistical analysis supports the primary role of irrigation in the historical increase of crop yield in India (Fig. 6a). We find that 638.8 ± 203.2 kg∙ha^-1^ of the crop yield increase is attributable to the growing irrigation practice, while CO_2_ fertilization and fertilizer use are responsible for 198.6 ± 160.8 kg∙ha^-1^ and 162.6 ± 226.4 kg∙ha^-1^ increase, respectively. Maximum temperature is identified as a negative stressor (–159.6 ± 56.1 kg∙ha^-1^), suggesting that recent warming and extreme heat events could result in a significant crop yield reduction^25,26^. The effectiveness of irrigation on crop yield enhancement is higher in drier regions compared to wetter environments confirming the MODIS-based sensitivity analysis presented in Fig. 3 (Fig. 6b). This varying level of irrigation effectiveness is also observable in a temperature gradient, i.e., the expanded irrigation on hotter regions has promoted greater yield increase compared to cooler regions.

**Fig. 7:**
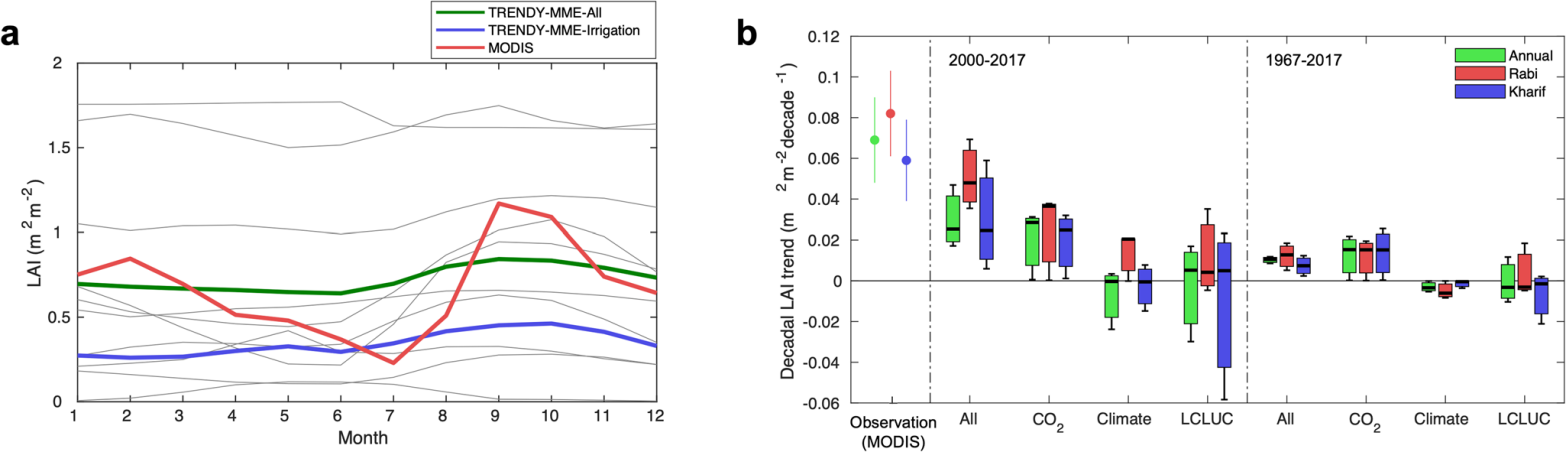
Comparison between satellite-derived and simulated seasonal LAI variation and long-term trends. **a** Seasonal LAI variation of MODIS (red) and TRENDY simulations (S3) (ensemble mean of all Dynamic Global Vegetation Models (DGVM): green, ensemble mean of all DGVMs accounting irrigation as land-use management (namely CLM5.0, LPJ-GUESS, and SURFEX): blue, individual models: light gray). **b** Annual (green) and seasonal (Rabi: red, Kharif: blue) leaf area index (LAI) trends derived from satellite observation (MODIS, circle) and simulated DGVMs (All, boxplot). Note that only DGVMs incorporating an irrigation component are included in this plot (see Supplementary Fig. S4 for the DGVMs without incorporating irrigation as a land management practice). The trends inferred from the DGVMs are attributed respectively to rising CO_2_, climate change, and land cover/use change (LCLUC) from factorial simulations. Two sets of LAI trend attribution results are presented for the periods of MODIS (2000−2017) and national statistics (1967−2017) records. Note that only DGVMs accounting for irrigation are considered in this comparison. The box stretches from the 25th percentile to the 75th percentile of all DGVMs. The median and mean values are shown as the solid and dot lines, respectively. Only cropland is considered in this comparison.

We further analyze the simulation results from the dynamic global vegetation models (DGVMs) that participated in the project named "Trends and drivers of the regional-scale sources and sinks of carbon dioxide" (TRENDY)^27^. Our examination aims to assess how well the state-of-art land surface models capture the observed dry season greening in India and how they attribute the LAI changes to different underlying drivers, i.e., CO_2_ fertilization, climate change, and LCLUC (Fig. 6). This analysis identifies three discernable differences between observations (satellite and national statistics) and model simulations. First, the irrigation-driven double cropping system (dual peaks, Fig. 2a) is not properly reproduced by TRENDY DGVMs (Fig. 7a). Second, the simulated annual LAI changes (mean ± std of models: 0.203 ± 0.145 m^2^ ∙ m^-2^∙decade^-1^) in India during the MODIS era (2000–2017) are two times smaller than the observed MODIS LAI change (0.069 ± 0.021 m^2^ ∙ m^-2^∙decade^-1^, *p* < 0.001) though inter-model variations exist (Fig. 7b). The magnitude of the simulated annual LAI changes during the longer-term period (1967–2017) is even lower than that observed for the MODIS era, which is inconsistent with the comparable degree of crop yield changes observed in both periods. Note that the trends of crop yield during the periods 1967–2017 and 2000–2017 are 293.5 ± 13.0 kg∙ha^-1^∙decade^-1^ and 340.0 ± 76.6 kg∙ha^-1^∙decade^-1^, respectively. Third, factorial attribution analysis of the TRENDY DGVM results identifies CO_2_ fertilization as the primary driver of Indian greening whereas climate and LCLUC have marginal impacts on the LAI changes. Though the TRENDY DGVMs presented in Fig. 7b account for irrigation practice (see Supplementary Fig. 4 for the TRENDY DGVMs without considering irrigation as a land-use management), the models identify the CO_2_ fertilization effect is a primary driver for the simulated greening in India. The CO_2_ fertilization effect is more prevalent in the long-term simulation result while climate and LCLUC have mostly negative effects on the LAI change. Yet, our results from MODIS and national agriculture statistics recognize the significant role of land management (i.e., primarily irrigation) in the observed greening and yield enhancement, while acknowledging a non-negligible role of CO_2_ fertilization effect on crop yield increase (Fig. 6a). Note that the total effect of irrigation on the historical crop yield changes is 3.2 times larger than the one of CO_2_ fertilization effect. These all collectively suggest that the TRENDY DGVMs tend to underestimate LCLUC contribution but overestimate CO_2_ fertilization effect on cropland greening in India.

## Discussion

The advantage of using time series of satellite records, compared to coarser scale grain production statistics, is that they can show details of spatial and temporal patterns of vegetation changes in croplands (Figs. 1 and 2)^16^. The observed patterns of seasonally resolved LAI change and its association with irrigation practices confirm the importance of human land management in the observed global greening phenomenon^6^. Particularly, the dry (Rabi) season greening and crop yield enhancement in India are significant. Earlier studies appreciated the importance of irrigation in the observed changes in greenness in India, but they often neglected the seasonally varying patterns of the changes^6,19^. This overlooked aspect is one of the important findings in this study, which suggests a continuous shift in India’s agriculture toward an irrigation-driven dry season cropping system. Agricultural production is governed by many economic, technological, and environmental factors. Thus, determining the precise contribution of each factor is generally extremely difficult and beyond the scope of this study. There are several other factors that could also be involved in the observed greening and increasing grain production that have not been considered in this study. For instance, fertilizer use, and mechanization could also be playing a significant role in the recent greening in India^28^. Nonetheless, without discounting the other contributing factors, this study clearly suggests a strong contribution of irrigation to the crop yield enhancement and greening trend in India by providing consistent evidence from independent multi-scale datasets.

This study clearly suggests that human land management activities have been important drivers for landscape greening and crop yield enhancement. However, the TRENDY DGVMs identify CO_2_ fertilization as a primary driver of increasing LAI over India and find climate and LCLUC as marginal or even negative contributors (Fig. 7b). This result from the TRENDY factorial simulations is well in accordance with the relevant previous studies, including TRENDY, Multi-scale Synthesis and Terrestrial Model Intercomparison Project (MsTMIP), Coupled Model Intercomparison Project Phase 5 (CMIP5), Coupled Model Intercomparison Project Phase 6 (CMIP6)^8–10,29^, but counter to our findings. According to the national statistics, Indian crop yield has increased by 327 % compared to the initial measure in the 1960s with changing atmospheric CO_2_ levels from 322.2 ppm to 408.7 ppm (+86.5 ppm) during the last five decades. Previous studies have reported that CO_2_ fertilization effect on crop yield per 100 ppm CO_2_ increase can range from 1 to 17% by regions and from 0 to 33 % by crop types^30,31^. Also, long-term free‐air CO_2_ enrichment (FACE) experiments have shown that elevation of CO_2_ by ca. 200 ppm causes a ca. 18% increase (ca. 9% per 100 ppm) in yield under non‐stress conditions^32^. Given survey-based statistics and previously reported CO_2_ fertilization effect estimates, the CO_2_ fertilization effect alone may not explain such significant upward trends in Indian crop yield during the last five decades. Our statistical analysis also supports that the increase of atmospheric CO_2_ concentration has positive effects on the historical crop yield enhancement but is not a primary driver as the models simulate (Fig. 6a). Such a large discrepancy between the model simulation and empirical investigations implies that the current DGVMs may have limited capability to realistically reflect processes of land use and management, and thus correctly attribute the LAI changes to the underlying drivers^33,34^. Another explanation of the discrepancy we find from the comparison between the simulated and MODIS LAIs is a potential issue in human-management forcing data. Our complementary analysis shows that Land-Use Harmonization (LUH) Version 2 which was used for LCLUC forcing in the TRENDY gives 18.6 ± 2.0 % less irrigated croplands in India (Supplementary Fig. 7). We also find that the change rate of percent of irrigated area inferred from LUH and the national statistics quite differs. The rate of change in the last two decades (2000–2018) from the national statistics is about 3.4 ± 0.7 %·decade^-1^ but LUH-based change rate (1.5 ± 0.6 %·decade^-1^) is half of the actual changes in India though its changing rate (2.9 ± 0.1 %·decade-1) in earlier period (1967–1999) is greater than the survey-based statistics (2.3 ± 0.3 %·decade^-1^). These limitations may explain why we see different LAI seasonality and greening patterns in the Rabi and Kharif seasons from the MODIS and the DGVMs (Fig. 7).

This finding holds significant implications for interpreting the large-scale leaf area increase and its consequences for Earth’s carbon, water, and energy cycles. For instance, the enhanced seasonal amplitude of atmospheric CO_2_ concentration during the last decades has been proposed to be a result of vegetation growth stimulated by higher concentrations of CO_2_, as well as by changing climate^35,36^. However, these mechanisms have proven insufficient in explaining the full range and magnitude of the observed increase in seasonal CO_2_ amplitude. An alternative hypothesis is that the intensification of agriculture through human land management primarily contributes to the seasonal changes in CO_2_ exchange between the biosphere and the atmosphere^37,38^. Extensive greening across global croplands further underscores the significance of our findings^6,13,14^, thereby providing additional evidence in support of the alternative hypothesis. Another important implication of our findings relates to strategic planning for mitigating climate change^39,40^ and ensuring food security^41^. Our results suggest that the expected response of vegetation to rising atmospheric CO_2_ levels may be smaller than previously thought, indicating that the carbon sequestration capacity of terrestrial ecosystems could be less significant than earlier estimates. This has critical implications for our current climate change mitigation strategy, which is built upon prior understanding. Furthermore, the potential costs associated with ensuring food security could be higher than previously anticipated. This is because the reduced crop yield enhancement from CO_2_ fertilization implies the need for significant additional land management to sustain the continuous increase in crop yield and production. These collectively underscore the previously overlooked role of human land use and management in global vegetation changes and highlight its potential implications across various Earth systems, including the human system.

Human land use and management has emerged as an important process in the earth system modeling framework as not only it has decisive impacts on the Earth system but also it can be a tool to mitigate global climate change^7^. However, as reported in this study, land management (here irrigation) has not been well incorporated into the land surface models^34^. A few of the TRENDY DGVMs used in this study (namely CLM5.0, LPJ-GUESS, and SURFEX) incorporate an irrigation practice as a process of land management (Supplementary Table 2), however, the observed irrigation-driven dry season greening is not reproduced by these models (Supplementary Fig. 3). Progress has been slow in incorporating land use and management processes into the earth system modeling frameworks, often limited by technical and data availability challenges^34^. For instance, information on soil management, crop varieties, crop rotations, and actual irrigation amounts and schemes is presently either not available or only incompletely so. It is also challenging to acknowledge and address errors in various processes, such as gross primary production, respiration, allocation of photosynthate, soil dynamics, and crop stress response, which can compensate for each other in the formation of yield and leaf area. These challenges suggest further model development and improvement, thereby urging a continuous and closer collaboration among the modeling, Earth observation, and land system science communities to better represent land use and management in LSMs.

Continuous irrigation practice and yield enhancement have raised an important concern regarding its sustainability because the observed irrigation-induced greening can have discernable impacts on India’s groundwater^42,43^. India is the world’s largest consumer of groundwater and groundwater provides ~60% of the nation’s irrigation supply. Recent studies have found that the intensified irrigation in India depletes groundwater^44^. Current trends in groundwater depletion in India are becoming a threat to food security because it leads to a decrease of cropping intensity by 20% nationwide and by 68% in groundwater-depleted regions^42^. The projected worsening of water resources in India suggests that the observed dry season cropping/greening will become increasingly susceptible to interannual rainfall variability, potentially leading to its diminishment. It thus becomes more uncertain to what extent and how long the observed Indian greening lasts under current groundwater depletion rates and changing climate. Similar situations may arise in other observed greening hotspots over the global breadbaskets as emerging studies point to challenges in water management in these regions^45,46^. This suggests that continuous monitoring of cropland greenness (or yield) is essential for sustainable water resource management and ensuring national and global food security.

## Methods

### MODIS LAI

The latest version (Collection 6, C6) of NASA Terra and Aqua MODIS LAI products (MOD15A2H and MYD15A2H) is used in this study^47,48^. These LAI datasets (2000–2018) are provided as 8-day composites with a 500-m sinusoidal projection. The datasets are refined by rigorous checking of the quality fags of the LAI products and of the simultaneous vegetation index products, following the previously described methods^49^. This filtering provides the highest quality MODIS LAI observations that minimize any residual contamination from clouds, aerosols, snow, and shadow 6. The two LAI datasets (that is, four 8-day composites) are then combined into a 16-day composite by taking the mean of all valid LAIs (temporal average). The quality of C6 MODIS LAI datasets has been comprehensively evaluated against ground-based measurements of LAI and through inter-comparisons with other satellite LAI products^50,51^. These datasets represent the latest and highest-quality LAI products that are currently available. This study uses the time series of 19-year MODIS LAI data averaged over the Rabi (November–May), Kharif (June–October), and annual time period.

### Indian agriculture statistics

Spatially aggregated historical annual and seasonal (Rabi and Kharif season) food grain production, yield, cropping area, and irrigation statistics are extracted from the Ministry of Agriculture, Government of India^52^. The data from 1967 to 2018 is used in this study. Food grain production refers to the total production of rice, wheat, corn, coarse grains (sorghum and millet), and pulses (beans, dried peas, and lentils). Crop-specific data for major crops (rice, wheat, pulses, maize, jowar, bajra, and nutrient cereal) is also prepared to investigate how individual crop’s yield, cropping area, total production, and irrigation statistics have changed differently during the last five decades.

### Cropland fraction, irrigation, and crop type map

We define the geographic distribution of croplands based on both MODIS Land Cover product (MCD12Q1) and International Institute for Applied Systems Analysis (IIASA) cropland fraction data^53^. In this study, we only keep pixels that satisfy two following conditions as croplands: (a) MCD12Q1 equals 12 (Croplands) or 14 (Cropland/Natural Vegetation Mosaics), (b) IIASA cropland fraction > 50 %. We also define the irrigated and unirrigated (i.e., rainfed) croplands in India by overlaying the global irrigated and rainfed cropland area maps^18^. We further introduce seasonally resolved crop type maps to understand to what extent the seasonally varying degree of changes in Indian cropland is associated with different crop types^54^. These crop-type maps were developed using MODIS 250 m surface reflectances (and derived spectral indices) and quantitative spectral matching techniques, resulting in mapped accuracies ranging from 72% to 97%. The crop types derived from remote sensing explained variability in national statistics ranging from 63% to 98%.

### Aridity and climate data

Aridity is usually expressed as a generalized function of precipitation, temperature, and reference evapotranspiration. It is considered an indicator of quantified precipitation availability over atmospheric water demand. The aridity index used in this study was calculated as a ratio between mean annual precipitation and reference evapotranspiration^55^. The precipitation data was obtained from the high-resolution WorldClim2 data while the reference evapotranspiration was modeled using the FAO Penman-Monteith method. In this study, we employed the UNEP aridity classification scheme: arid (<0.2), semi-arid (0.2–0.5), dry sub-humid (0.5–0.65), and humid (>0.65). For assessing relations between MODIS LAI and climate data, we obtained monthly temperature, precipitation, and radiation data from CRU TS (Climatic Research Unit gridded Time Series) Version 4.04 data^56^.

### TRENDY DGVM LAI

DGVMs simulate key physical and biological processes of the land system in interaction with the atmosphere. DGVMs provide a deeper insight into the mechanisms controlling terrestrial energy, hydrological, and carbon cycles, as well as the drivers of phenomena ranging from short-term anomalies to long-term changes^57^. DGVM simulations under constant environmental conditions have been performed within the project TRENDY (Trends and drivers of the regional-scale sources and sinks of carbon dioxide)^27^. In this study, we use 10 TRENDY v7 DGVMs including CABLE-POP^58^, CLM5.0^59^, JSBACH^60^, JULES^61^, LPJ-GUESS^62^, LPX-Bern^63^, ORCHIDEE^64^, SDGVM^65^, SURFEX^66^, and VISIT^67^ (Supplementary Table 1). A set of three experiments driven by either constant or varying climate data and other inputs such as atmospheric CO_2_ and LCLUC forcing were designed in the TRENDY project to differentiate the role of CO_2_, Climate, and LCLUC (Supplementary Table 2). The TRENDY v7 models were forced by gridded climate data (either monthly CRU or 6-hourly CRU-JRA55), atmospheric CO_2_ concentrations based on ice core measurements (pre-1958), and stationary observations from the National Oceanic and Atmospheric Administration (NOAA) (post-1958), and Land-use Harmonization (LUH) Version 2 data^68^. The TRENDY models provided three types of simulations: (a) one that considers the variability in atmospheric CO_2_ (S1), (b) one that considers the variability in CO_2_ and climate (S2), and (c) one that considers the variability in CO_2_, climate, and historical LCLUC (S3). We aggregate the DGVM simulated monthly LAIs to the Rabi (November–May), Kharif (June–October), and annual LAI time series.

### Analytical approaches

We evaluated each LAI time series (Rabi, Kharif, and annual LAI) for the presence of a monotonic trend using a rank-based Mann–Kendall trend test^69^ and determined the slope of each time series using a non-parametric Theil–Sen slope estimator^70^ as implemented using the zyp package^71^ in R. This approach for robust trend assessment accounts for potential temporal autocorrelation and has been used in prior studies that evaluated changes in target variables including remote sensing and climate variables 6. We classified pixels (or aggregated time series) with a positive LAI trend (*p* < 0.1) as greening or a negative LAI trend (*p* < 0.1) as browning.

The role of irrigation in modulating leaf area changes is evaluated using irrigated and unirrigated area maps^18^. We also further categorize croplands based on aridity levels (arid, semi-arid, dry sub-humid, and humid) to investigate the role of irrigation in the satellite-observed greening across varying aridity levels. We compare trend estimates between different irrigation practices and/or aridity conditions. In this study, we further apply a partial-correlation analysis to evaluate the responsiveness of MODIS LAI to precipitation variability after statistically controlling for the covarying effects of MODIS LAI and climatic variables (temperature and solar radiation). These climatic variables are all derived from the CRU TS4.04 datasets.

We quantify trends in total production, yield, cropping area, and irrigation statistics of both food grain and major crop types (rice, wheat, pulse, maize, jowar, Bajra, and nutrient cereal) in India, and evaluate relations between each variable using Pearson correlation coefficient. In this analysis, we further split the statistics into two time periods (Earlier 20 years: 1967–1986, Later 20 years: 1999–2018) to investigate how the trends and correlations evolve in the earlier and later periods.

We also use multivariate regression analysis to quantify the effect size of irrigation, fertilizer, atmospheric CO_2_ concentration, and climate (temperature, precipitation, and radiation) on historical crop yield changes. Time series of the dependent and independent variables are prepared for this analysis. Given the limited availability of historical fertilizer use records, we only use the data from 1985 to 2017 for this analysis. To further investigate interactions between irrigation practice and climate factors, we include interaction terms, i.e., irrigation × precipitation (ppt) and irrigation × maximum temperature (tmax). Our general model of crop yield, which includes 5 covariates and 2 interaction terms, is:

$${{{{{\mathrm{Yield}}}}}} \sim \,	 {{{{{\mathrm{Irrigation}}}}}}+{{{{{\mathrm{Fertilizer}}}}}}+{{{{{{\mathrm{CO}}}}}}}_{{{{{\mathrm{2}}}}}}+{{{{{\mathrm{ppt}}}}}}\\ 	+{{{{{\mathrm{tmax}}}}}}+{{{{{\mathrm{rad}}}}}}+{{{{{\mathrm{Irrigation}}}}}} \! \! :{{{{{\mathrm{ppt}}}}}}+{{{{{\mathrm{Irrigation}}}}}} \! \! :{{{{{\mathrm{tmax}}}}}}$$

To quantify the individual contribution of CO_2_, climate, and land cover/use changes (LCLUC) to changes in LAI, we follow a factorial simulation approach using three different simulations of DGVMs in TRENDY^27,72^. The effect of CO_2_ on the LAI change is represented by a trend of S1 (CO_2_ only) results; the S2 (CO_2_ + Climate) results show a trend that is the sum of CO_2_ and climate effects, and the S3 (CO_2_ + Climate + LCLUC) simulations include trends from time-varying CO_2_, climate, and land use/cover change. For simplicity, the effect of “climate” as used in this paper includes the synergy of CO_2_ and climate, and similarly the effect of “LCLUC” also includes the synergy terms. Therefore, the effects of CO_2_, climate, and LCLUC are then quantified as the trend for S1, the trend of S2 minus the S1 trend, and the trend of S3 minus the S2 trend, respectively. All trends are evaluated by the non-parametric Theil–Sen slope estimator^70^.

### Supplementary information

Supplementary Material

## Data Availability

MODIS LAI (MOD15A2H & MYD15A2H) and LC (MCD12Q1) data datasets are available from the NASA Earth Observing System Data and Information System (https://earthdata.nasa.gov/). Agriculture statistics is available at https://agricoop.gov.in/en/Agricultural_Statistics_at_a_Glance. Cropland irrigation and crop type maps are available at https://doi.pangaea.de/10.1594/PANGAEA.884744 and http://maps.icrisat.org/. TRENDY DGVM data is available at https://blogs.exeter.ac.uk/trendy/. WorldClim2 and CRU data are available at https://www.worldclim.com/version2 and https://crudata.uea.ac.uk/cru/data/hrg/, respectively.

## Code availability

The code developed for the processing and analysis of data and to generate figures and tables in this analysis is available from the corresponding author upon reasonable request.
