# Supplementary material for: Greening of human-dominated ecosystems in India
Source: Commun Earth Environ. 2023 Nov 27;4(1):419. doi: 10.1038/s43247-023-01078-9 (PMC11041707; doi:10.1038/s43247-023-01078-9)
Supplement: Supplementary file 1 — Supplementary Material [file 43247_2023_1078_MOESM1_ESM.pdf]

Supplementary information

Greening of human-dominated ecosystems in India

Taejin Park[1,2], Murali K. Gumma[3], Weile Wang[1], Pranay Panjala[3], Sunil K. Dubey[4], Ramakrishna R. Nemani[1,2]

[1]NASA Ames Research Center, CA, United States
[2]Bay Area Environmental Research Institute, CA, United States
[3]International Crop Research Institute for Semi-Arid Tropics, Telangana, India
[4]Mahabalonis Crop Forecasting Center, Pusa Campus, New Delhi, India

Supplementary Tables (4)
Supplementary Figures (7)

Supplementary Table 1 | Overview of the TRENDY v7 DGVM models and their key processes relevant for the LCLUC.

Model	Irrigation	Fertilization	Cropland harvest
CABLE-POP[41]	No	No	Yes
CLM5.0[42]	Yes	Yes	Yes
JSBACH[43]	No	No	Yes
JULES[44]	No	No	No
LPJ-GUESS[45]	Yes	Yes	Yes
LPX-Bern[46]	No	Yes	Yes
ORCHIDEE[47]	No	Yes	Yes
SDGVM[48]	No	No	Yes
SURFEX[49]	Yes	No	No
VISIT[50]	No	No	Yes

Supplementary Table 2 | Overview of the simulations used in our study.

Simulation	Climate	CO_2	LCLUC
S0	Pre-industrial	Pre-industrial	Pre-industrial
S1	Pre-industrial	Observed	Pre-industrial
S2	Observed	Observed	Pre-industrial
S3	Observed	Observed	LUH2/HYDE

Supplementary Table 3 | Trends (per decade) in production, yield, cropping area, and irrigation statistics of major crop types in India. Three different periods for trend analysis were performed for seven major crop types: 1967-2018 (entire period), 1967-1986 (first 20 years), and 1989-2018 (last 20 years). The significance of the slope estimate is denoted by double ($p < 0.001$) and single ($p < 0.05$) asterisks.

Variable	Trend Period	Trend estimate (per decade)						
		Rice	Wheat	Pulse	Nutrient Cereal	Maize	Jowar	Bajra
Production (MT)	Entire period	15.17**	16.39**	1.37**	3.12**	3.40**	-1.16**	0.96**
	First 20 years	13.38**	16.87**	1.01*	2.33*	0.90*	1.53*	0.25
	Last 20 years	14.71**	16.80**	4.24**	7.89**	8.48**	-1.76**	1.26*
Yield (Kg/ha)	Entire period	307.28**	448.45**	52.67**	210.29**	347.64**	81.67**	169.38**
	First 20 years	250.67**	489.67**	18.40	110.00**	126.68*	125.51*	55.23
	Last 20 years	324.72**	339.86**	115.49**	441.46**	584.67**	78.93	301.63**
Area (Mha)	Entire period	0.16**	0.26**	0.03*	-0.47**	0.07**	-0.27**	-0.09**
	First 20 years	0.28**	0.50**	0.09*	-0.35**	0.01	-0.10*	-0.09*
	Last 20 years	-0.08	2.87**	2.54**	-3.31**	1.78**	-2.76**	-1.27*
Irrigation (%)	Entire period	5.30**	9.52**	2.25**	1.54**	1.96**	1.34**	1.17**
	First 20 years	3.00**	15.41**	-0.80*	-0.08	1.85*	0.27	1.16**
	Last 20 years	3.82**	4.49**	4.50**	3.70**	3.74**	1.20**	1.41*

Supplementary Table 4 | Relations between crop production, yield, cropping area, and irrigation. First and last 20 years of national statistics for seven major crop types were used for Pearson correlation analysis between production, yield, cropping area, and irrigation: 1967-1986 (first 20 years), and 1989-2018 (last 20 years). The significance of the slope estimate is denoted by double ($p < 0.001$) and single ($p < 0.05$) asterisks.

Variable	Period	Pearson correlation coefficient						
		Rice	Wheat	Pulse	Nutrient Cereal	Maize	Jowar	Bajra
Production vs. Yield	First 20 years	1.00^{**}	0.99^{**}	0.95^{**}	0.89^{**}	0.97^{**}	0.95^{**}	0.95^{**}
	Last 20 years	0.98^{**}	0.95^{**}	0.95^{**}	0.96^{**}	0.99^{**}	-0.01	0.81^{**}
Production vs. Area	First 20 years	0.95^{**}	0.95^{**}	0.66^{*}	-0.17	0.48^{*}	-0.16	0.48^{*}
	Last 20 years	0.36	0.91^{**}	0.94^{**}	-0.66^{**}	0.97^{**}	0.86^{**}	0.11
Yield vs. Irrigation	First 20 years	0.74^{**}	0.91^{**}	-0.43^{*}	-0.20	-0.06	0.68^{**}	0.03
	Last 20 years	0.88^{**}	0.75^{**}	0.80^{**}	0.90^{**}	0.87^{**}	0.73^{**}	0.17

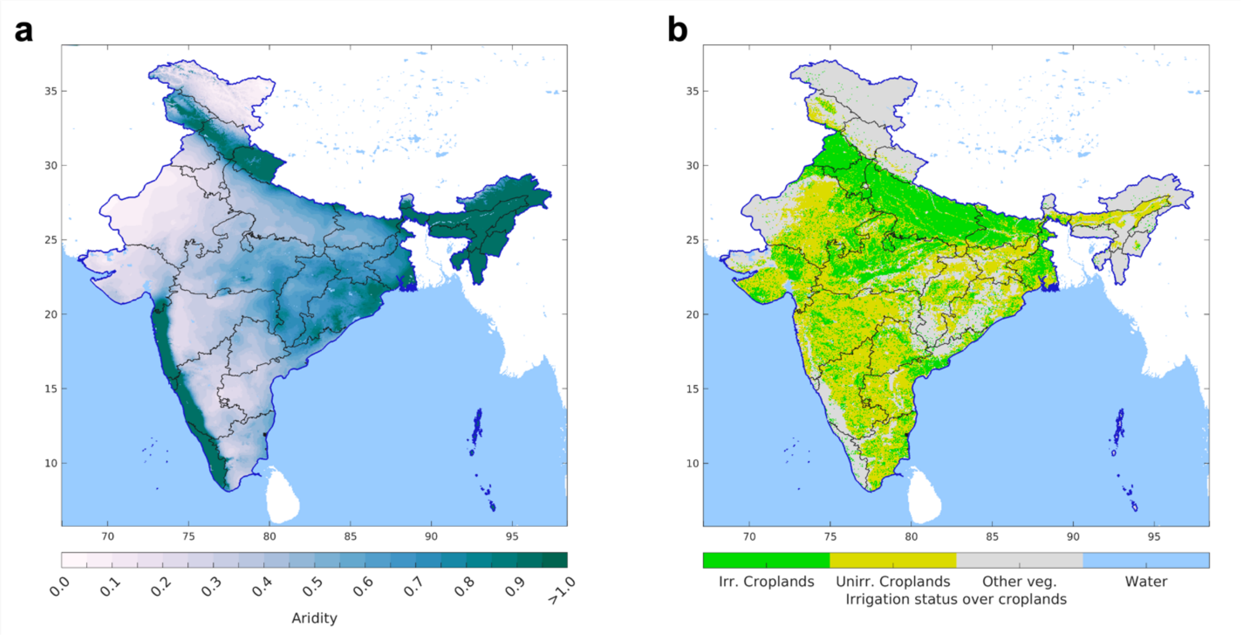

Supplementary Figure 1 | Spatial distribution of aridity and irrigation practice in India. a, Aridity. **b,** Irrigation distribution. Black and blue lines are state and country boundaries, respectively.

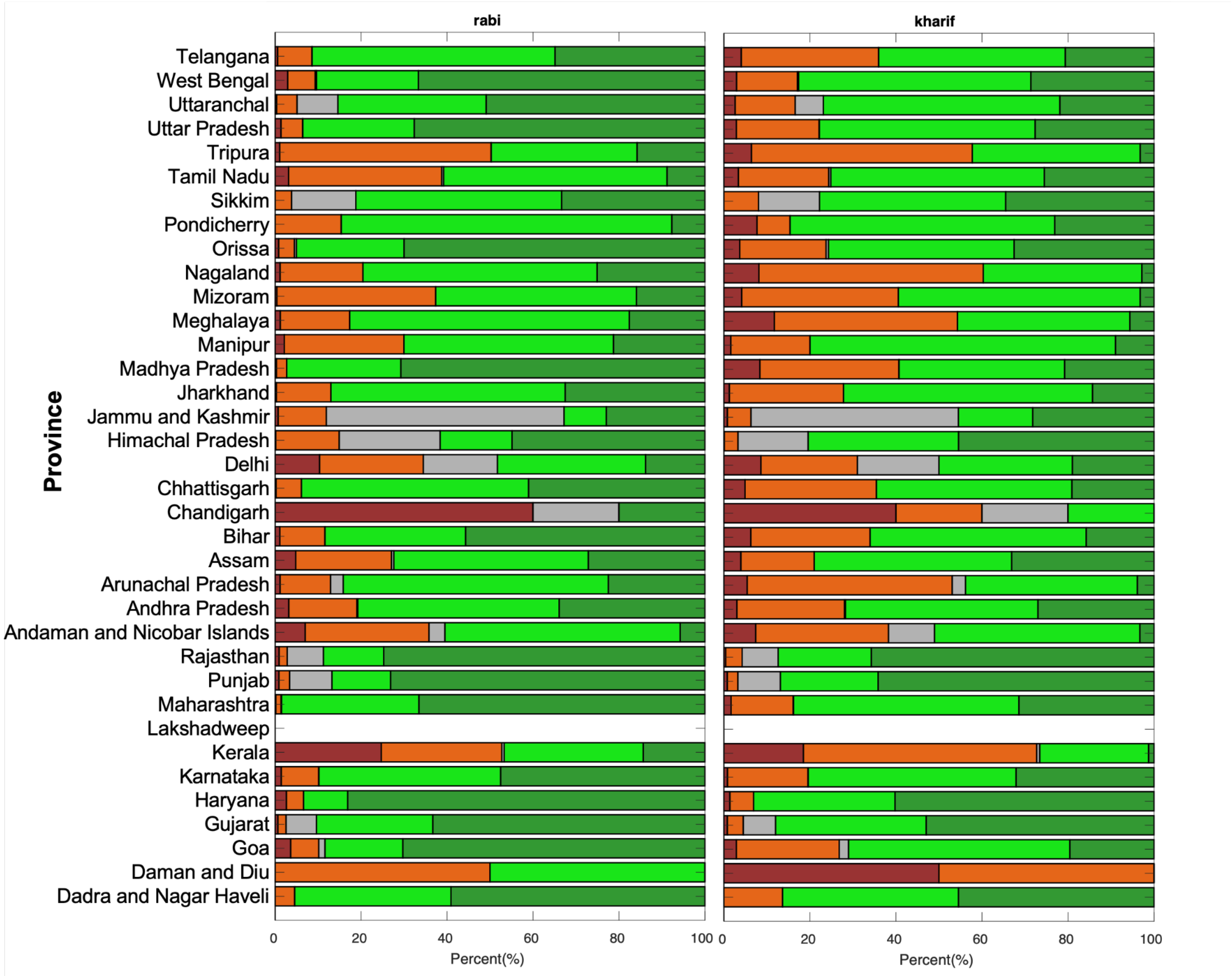

Supplementary Figure 2 | Percent of greening and browning across Indian provinces. Red, orange, gray, light green, and dark green stand for significant negative, insignificant negative, no change, insignificant positive, and significant positive trends. Here p-value 0.1 is used for defining statistical significant trends.

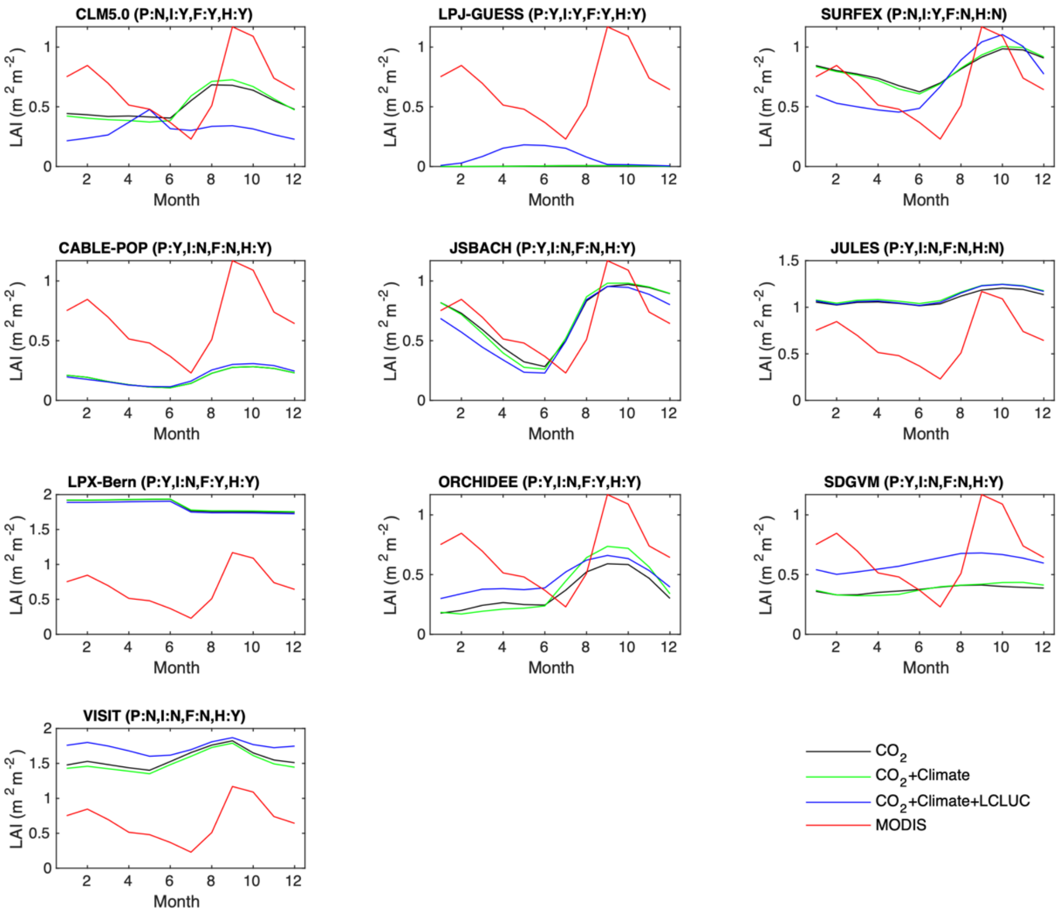

Supplementary Figure 3 | Comparison between satellite-derived and simulated seasonal LAIs from individual DGVMs. Seasonal LAI variation of MODIS (red) and TRENDY simulations (gray: S1, green: S2, and blue: S3).

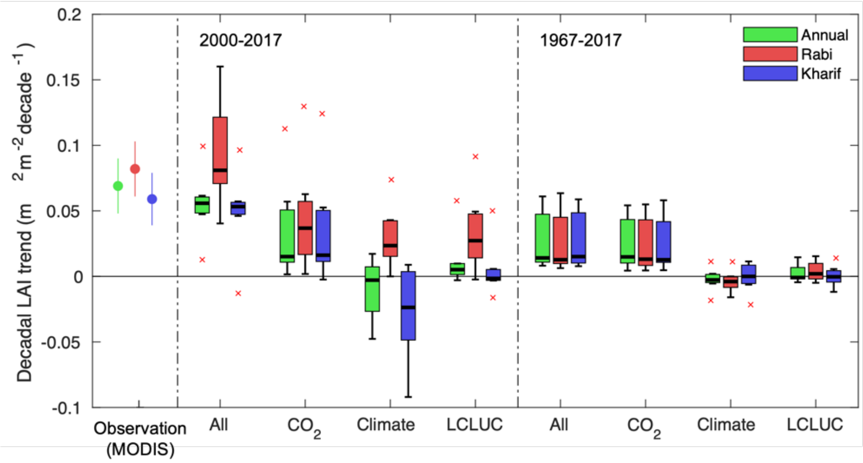

Supplementary Figure 4 | Comparison between satellite-derived and simulated long-term seasonal LAI trends. Annual (green) and seasonal (Rabi: red, Kharif: blue) leaf area index (LAI) trends derived from satellite observation (MODIS, circle) and simulated DGVMs (All, boxplot). Note that only DGVMs without incorporating an irrigation component are included in this plot. The trends inferred from the DGVMs are attributed respectively to rising CO_2, climate change, and land cover/use change (LCLUC) from factorial simulations. Two sets of LAI trend attribution results are presented for the periods of MODIS (2000-2017) and national statistics (1967-2017) records. Note that only DGVMs accounting irrigation are considered in this comparison. The box stretches from the 25th percentile to the 75th percentile of all DGVMs. The median and mean values are shown as the solid and dot line, respectively. Only cropland is considered in this comparison.

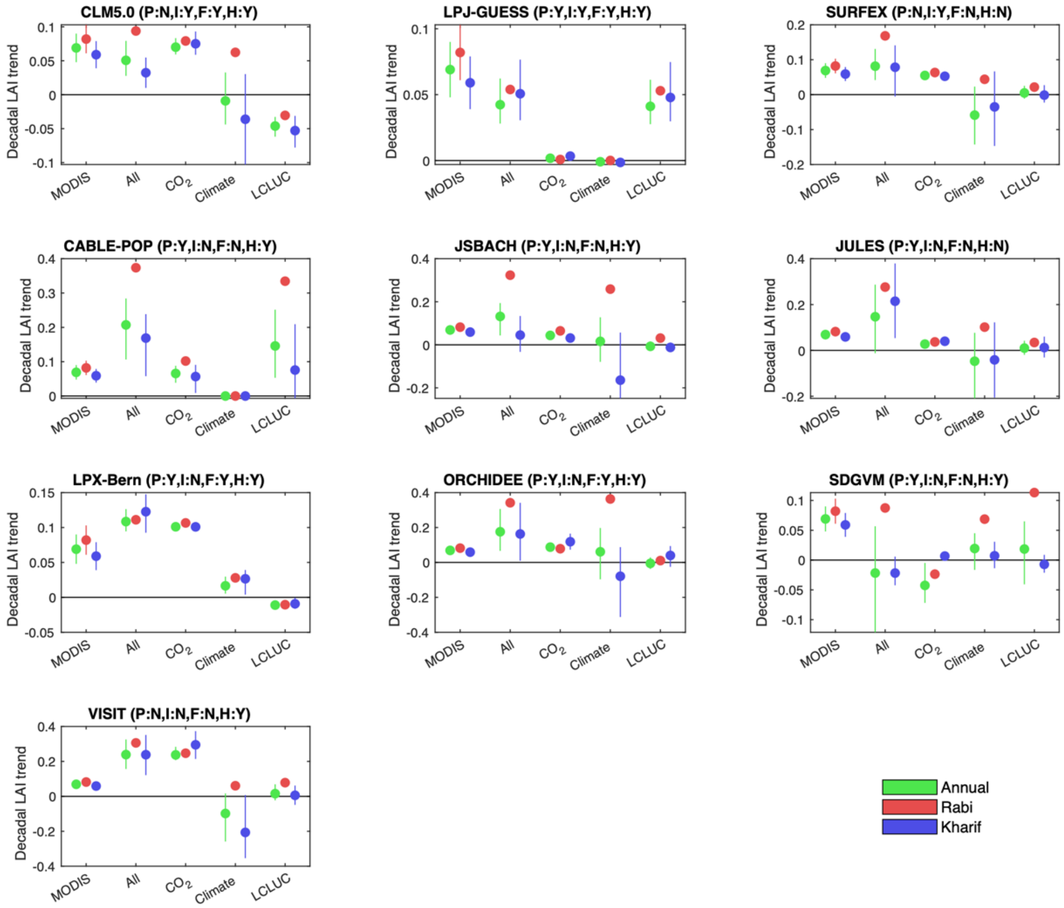

Supplementary Figure 5 | Comparison between satellite-derived (2000-2017) and simulated (2000-2017) long-term trends of seasonal LAIs from individual DGVMs. Annual (green) and seasonal (Rabi: red, Kharif: blue) leaf area index (LAI) trends derived from satellite observation and simulated DGVMs.

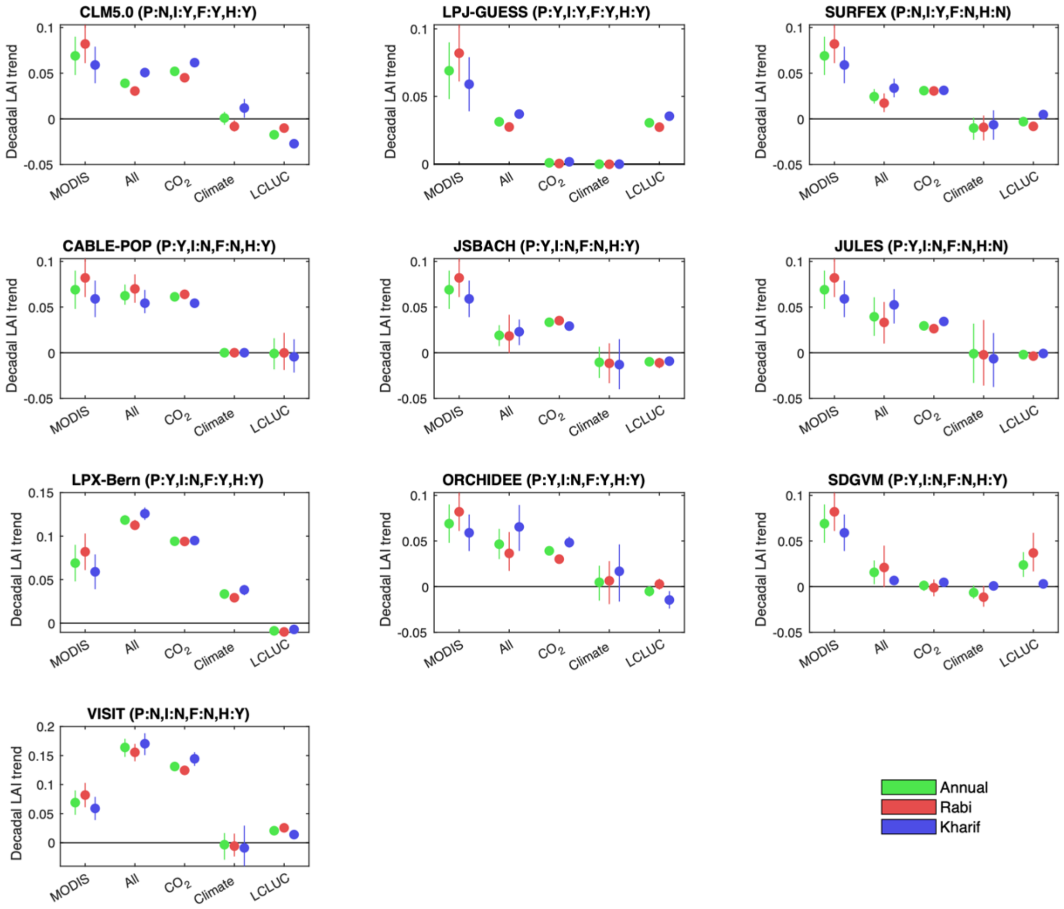

Supplementary Figure 6 | Comparison between satellite-derived (2000-2017) and simulated (1967-2017) long-term trends of seasonal LAIs from individual DGVMs. Annual (green) and seasonal (Rabi: red, Kharif: blue) leaf area index (LAI) trends derived from satellite observation and simulated DGVMs.

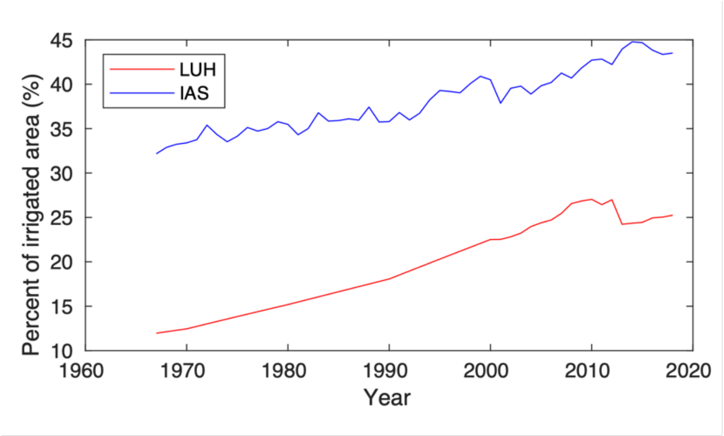

Supplementary Figure 7 | Changes in percent of irrigated area referred from national statistics and TRENDY LCLUC forcing. India's agriculture statistics (IAS, blue) and Land-Use Harmonization (LUH, red) Version 2 data which is used for LCLUC forcing in TRENDY simulation.
